# Supplementary material for: Empirical methods for controlling false positives and estimating confidence in ChIP-Seq peaks
Source: BMC Bioinformatics. 2008 Dec 5;9:523. doi: 10.1186/1471-2105-9-523 (PMC2628906; doi:10.1186/1471-2105-9-523)
Supplement: Additional File 1 — A variety of html documents from the USeq web site detailing the available applications, their best usage, output file type descriptions, command line menus, etc. [file 1471-2105-9-523-S1.zip › USeqUserGuides/JavaDoc.zip!/JavaDoc/trans/main/ScanChip.html]

ScanChip


|  |  |  |
| --- | --- | --- |
| **Overview** \| **Package** \| **Class** \| **Use** \| **Tree** \| **Deprecated** \| **Index** \| **Help** | |  |
| **PREV CLASS**   **NEXT CLASS** | **FRAMES**    **NO FRAMES**     **All Classes** |  |
| SUMMARY: NESTED \| FIELD \| CONSTR \| METHOD | DETAIL: FIELD \| CONSTR \| METHOD |  |


---


## trans.main Class ScanChip

```
java.lang.Object
  trans.main.ScanChip
```

---

``` public class ScanChip extends java.lang.Object ```

Scores windows of intensity values using several tests.

---

**Constructor Summary**

`ScanChip(java.lang.String[] args)`


| **Method Summary** | |
| --- | --- |
| `void` | `addPermutatedScoresToHistogram(java.util.ArrayList windowsAL)`             Uses random permutation of the cel file labels to estimate a null distribution, store random scores in a histogram for p-value and FDR estimations. |
| `void` | `buildExtensionSetThreshold()` |
| `void` | `calculateRandomLabelPermutationConfidenceScores()`             Takes the histogram of random permutation scores and calculates an uncorrected p-value and FDR for each real score. |
| `void` | `convertRatiosToPValues()` |
| `static float[][]` | `fetchFloatArrays(java.lang.String dir, boolean randomize)`             Given a directory (or a file), fetches serialized float[] from all files with the .celp extension. |
| `static java.lang.String[]` | `getSCORE_DESCRIPTION()` |
| `void` | `importScores(Window[] windows, java.io.File importFile)`             Reads in a file of scores from a qvalue output file Also writes and zips an .sgr file for the qValues. |
| `static void` | `main(java.lang.String[] args)` |
| `void` | `makeChromosomeWideScores()`             Makes chromosome wide scores. |
| `void` | `makeSgrFile(Window[] windows, java.io.File sgrFile, int scoreIndex)`             Makes a xxx.sgr.zip file from a sorted Window[]. |
| `static void` | `printDocs()` |
| `void` | `printOligoRatios()`             Prints log2 oligo ratios. |
| `void` | `processArgs(java.lang.String[] args)`             This method will process each argument and assign new varibles |
| `double` | `scoreWindow(int testWindowIndex)`             Scores a window using a pseudo median or trimmed mean on oligo relative differences. |
| `void` | `shuffleIntensities()`             Combines and shuffles the treatment and control intensities. |

**Methods inherited from class java.lang.Object**

`equals, getClass, hashCode, notify, notifyAll, toString, wait, wait, wait`

**Constructor Detail**

### ScanChip

```
public ScanChip(java.lang.String[] args)
```


**Method Detail**

### addPermutatedScoresToHistogram

```
public void addPermutatedScoresToHistogram(java.util.ArrayList windowsAL)
```

:   Uses random permutation of the cel file labels to estimate a null distribution,
    store random scores in a histogram for p-value and FDR estimations.

---


### shuffleIntensities

```
public void shuffleIntensities()
```

:   Combines and shuffles the treatment and control intensities.

---


### calculateRandomLabelPermutationConfidenceScores

```
public void calculateRandomLabelPermutationConfidenceScores()
```

:   Takes the histogram of random permutation scores and calculates an uncorrected p-value and FDR for each real score.

---


### scoreWindow

```
public double scoreWindow(int testWindowIndex)
```

:   Scores a window using a pseudo median or trimmed mean on oligo relative differences.
    If the number of relative differences exceed the maxScores then they are randomly
    sampled to the number of maxScores prior to running the pseudo median.
    Returns 0 if using pseudoMedian and window size is < 2 or if using trm meand and window size < 3.

    :   **Returns:**: trimmed mean average or pseudo median layered relative difference score -2 to 2.

---


### convertRatiosToPValues

```
public void convertRatiosToPValues()
```

---


### printOligoRatios

```
public void printOligoRatios()
```

:   Prints log2 oligo ratios.

---


### makeChromosomeWideScores

```
public void makeChromosomeWideScores()
```

:   Makes chromosome wide scores.

---


### importScores

```
public void importScores(Window[] windows,
                         java.io.File importFile)
```

:   Reads in a file of scores from a qvalue output file
    Also writes and zips an .sgr file for the qValues.
    Saves score in last position of the window.scores[].

---


### fetchFloatArrays

```
public static float[][] fetchFloatArrays(java.lang.String dir,
                                         boolean randomize)
```

:   Given a directory (or a file), fetches serialized float[] from all files with the .celp extension.
    Will randomize the float[]s if so indicated.

---


### main

```
public static void main(java.lang.String[] args)
```

---


### processArgs

```
public void processArgs(java.lang.String[] args)
```

:   This method will process each argument and assign new varibles

---


### printDocs

```
public static void printDocs()
```

---


### getSCORE\_DESCRIPTION

```
public static java.lang.String[] getSCORE_DESCRIPTION()
```

---


### buildExtensionSetThreshold

```
public void buildExtensionSetThreshold()
```

---


### makeSgrFile

```
public void makeSgrFile(Window[] windows,
                        java.io.File sgrFile,
                        int scoreIndex)
```

:   Makes a xxx.sgr.zip file from a sorted Window[].


---


|  |  |  |
| --- | --- | --- |
| **Overview** \| **Package** \| **Class** \| **Use** \| **Tree** \| **Deprecated** \| **Index** \| **Help** | |  |
| **PREV CLASS**   **NEXT CLASS** | **FRAMES**    **NO FRAMES**     **All Classes** |  |
| SUMMARY: NESTED \| FIELD \| CONSTR \| METHOD | DETAIL: FIELD \| CONSTR \| METHOD |  |


---
